# Patient responses to research recruitment and follow-up surveys: findings from a diverse multicultural health care setting in Qatar

**DOI:** 10.1186/s12874-016-0109-3

**Published:** 2016-01-26

**Authors:** Amal Khidir, Humna Asad, Huda Abdelrahim, Maha Elnashar, Amal Killawi, Maya Hammoud, Abdul Latif Al-Khal, Pascale Haddad, Michael D. Fetters

**Affiliations:** Pediatric Clerkship, Weill Cornell Medical College in Qatar, Education City, PO Box 24144, Doha, Qatar; Weill Cornell Medical College in Qatar, Olin Hall, Suite 432, 445 East 69 Street, New York, NY 10022 USA; Weill Cornell Medical College in Qatar, PO Box 24144, Doha, Qatar; The Center for Cultural Competence in Healthcare, Weill Cornell Medical College in Qatar, PO Box 24144, Doha, Qatar; Department of Family Medicine, University of Michigan, Ann Arbor, MI 48104-1213 USA; University of Michigan, 1500 E. Medical Center Drive, L4000 Women’s, Ann Arbor, MI 48109 USA; Department of Medical Education, Hamad General Hospital, PO Box 3050, Doha, Qatar; Biostatistics, Epidemiology and Biomathematics Research Core, Research Department, Weill Cornell Medical College in Qatar, Education City, PO Box 24144, Doha, Qatar; Department of Family Medicine, University of Michigan, 1018 Fuller Street, Ann Arbor, MI 48104-1213 USA; Mixed Methods Research and Scholarship Program, University of Michigan, 1018 Fuller Street, Ann Arbor, MI 48104-1213 USA; Japanese Family Health Program, University of Michigan, Japanese Family Health Program 24 Frank Lloyd Wright Dr., SPC 5795, Ann Arbor, MI 48106-5795 USA; Present address: Unit 42, 667 Pinerow Crescent, Waterloo, ON N2T 2L5 Canada; Present address: 435 Van Houten Ave. Apt #205, Passaic, NJ 07055 USA; Present address: 6 Rosenstrasse, 40479 Dusseldorf, Germany

**Keywords:** Recruitment, Mail survey, Follow- up, Cultural clues model, Validity testing, Reliability testing, Consent, Survey research

## Abstract

**Background:**

Health care researchers working in the Arabian Gulf need information on how to optimize recruitment and retention of study participants in extremely culturally diverse settings. Implemented in Doha, Qatar in 2012 with 4 language groups, namely Arabic, English, Hindi, and Urdu, this research documents persons’ responses to recruitment, consent, follow-up, and reminder procedures during psychometric testing of the Multicultural Assessment Instrument (MAI), a novel self- or interviewer-administered survey.

**Methods:**

Bilingual research assistants recruited adults in outpatient clinics by approaching persons in particular who appeared to be from a target language group. Participants completed the MAI, a second acculturation instrument used for content-validity assessment, and a demographics questionnaire. Participants were asked to take the MAI again in 2–3 weeks, in person or by post, to assess test-retest reliability. Recruitment data were analyzed by using nonparametric statistics.

**Results:**

Of 1503 persons approached during recruitment, 400 enrolled (27 %)—100 per language group. The enrollment rates in the language groups were: Arabic-32 %; English-33 %; Hindi-18 %; Urdu-30 %. The groups varied somewhat in their preferences regarding consent procedure, follow-up survey administration, contact mode for follow-up reminders, and disclosure of personal mailing address (for postal follow-up). Over all, telephone was the preferred medium for follow-up reminders. Of 64 persons who accepted a research assistant’s invitation for in-person follow-up, 40 participants completed the interview (follow-up rate, 63 %); among 126 persons in the postal group with a deliverable address, 29 participants mailed back a completed follow-up survey (response rate, 23 %).

**Conclusions:**

Researchers in the Arabian Gulf face challenges to successfully identify, enroll, and retain eligible study participants. Although bilingual assistants—often from the persons’ own culture—recruited face-to-face, and our questionnaire contained no health care-related content, many persons were reluctant to participate. This occurrence was observed especially at follow-up, particularly among participants who had agreed to follow-up by post.

## Background

Although countries in the Arabian Gulf and Middle East have in recent years gained geopolitical prominence in the international arena, they have been all but ignored in the literature on participant recruitment in research studies. Cities such as Doha, in Qatar, and Dubai, in the United Arab Emirates, along with countries like Bahrain and Kuwait, have extremely high-density, multicultural populations [1]. In these extraordinarily diverse communities, large numbers of individuals from different cultures and backgrounds live and work side-by-side, including many expatriate workers recruited as common laborers, who often have low levels of literacy.

Previously, researchers in various settings have examined methods of obtaining consent for study participation, compensation for study enrollment or follow-up, and preferred modes of administration and reminders for follow-up surveys [2–17]. However, little research into these aspects of recruitment and participation has been conducted in the multicultural, multilingual settings of the Gulf countries, the Middle East more generally, or Arab or Muslim communities [18–32]. Moreover, no identifiable study in these communities has involved the use of the postal system for collecting data, conducting follow-up or issuing follow-up reminders.

In general, relatively few studies have compared participation and response rates by mail or e-mail versus in-person, face-to-face surveys or interview-based research [7, 8, 10–12, 33, 34]. Most such studies have compared mail with e-mail [8, 10–12, 34]; few have compared mailed surveys with in-person administration [7, 14]. The response rates have varied depending on participants’ interest level in the topic, the perceived relevance and sensitivity of the topic, the types of questions asked [14, 32], and the participants surveyed (professionals vs. members of the general public). Anonymity and compensation have some effect on participation in some studies [2, 6], but not in others [4, 6, 15]. Compared with mailed surveys, follow-up in person is considered to be more expensive and more likely to lead to misinterpretation and biased responses; in contrast, follow-up by post is considered to be cheaper and more anonymous [7, 14]. E-mail is faster than post [8, 10, 11]. Response rates by post have been relatively high in some studies but have less so in others [8, 10, 32, 34]. Olson et al. found that the preferred survey mode might vary among participants, although there was no clear evidence that using participants’ preferred modes affected the response rate [33]. In the same study, interview by home phone was preferred by 49 % of participants, mailed questionnaire by 25 %, and Web survey by 20 %. In another study, the response rate by mail was higher even if it was not the preferred mode, although the overall rate increased by giving participants a choice [14].

Given its leadership role in the Arabian Gulf, Qatar is an excellent setting for investigating approaches to recruitment procedures. To date, no published study has compared response rates of surveys conducted in person versus by mail, nor has any study reported participants’ preferred procedure of data collection or preference for reminders in Qatar or the Middle East. Few studies suggest strategic methods to help recruit participants in a multicultural context [26, 35]. One generally useful strategy is to hire research assistants (RAs) who reside in-or have built trust with-the community, or who have the same cultural background as the potential participants [2, 3, 5, 23, 25, 27, 29]. However, there is no guide or published information available to help researchers identify particular linguistic, ethnic, or other groups or subgroups for recruitment among an extremely diverse patient population. The literature on obtaining informed consent in the Middle East is limited [26], and no known published study has compared the use of in-person interviews versus mailed questionnaires at follow-up for survey reliability testing.

As the world’s third-largest natural gas producer, Qatar has a rapidly transforming economy, with notable gains in its education and research sectors. Moreover, it is like a mosaic due to the extremely diverse population [1]; Qatari nationals make up only 6 % of the country’s workforce, while non-Qataris constitute 94 % [36]. The majority of expatriates in Qatar fall into one of two groups: highly educated professionals or low-literacy or illiterate laborers, for the latter, especially within construction jobs. Moreover, 75 % of non-Qatari workers are semiskilled or unskilled, and 50 % of expatriate workers have no more than a primary school education.

Community-based research and research networks are not yet well-established in Qatar; moreover, research awareness is relatively new in this highly linguistically and culturally diverse society [35]. Understanding the pros and cons of different recruitment strategies is particularly important in Qatar, where postal addresses are mainly work addresses or personal post office (PO) boxes. Few people use a home street address for mailing purposes [37].

Given the mosaic population in Qatar, the researchers sought to develop a robust measure of acculturation, the Multicultural Assessment Instrument (MAI). Development and testing of this novel instrument is part of a bigger multistage investigation funded by the Qatar National Research Fund to develop a health care quality-assessment instrument in the four most commonly spoken languages in Qatar: Arabic, English, Hindi, and Urdu [26, 35]. The overarching theoretical framework for the entire multistage mixed methods investigation was the “cultural construction of clinical reality” model originating from the work of medical anthropologist Arthur Kleinman [38, 39]. The project was designed to consider the structural domains of health care related to the professional, the popular, and folkways. This framework compelled us to develop an instrument to assess acculturation appropriate for Qatar and the Arabian Gulf. The research team knew that in Qatar, with its extremely diverse population of expatriate workers, identifying eligible participants would be a challenge. In particular, primary English speakers have vastly different cultural backgrounds. One authority based on extensive research experience estimates the English speaking population to have origins from Europe (25 %), the Philippines (15 %), Indian subcontinent (15 %), and Arabic countries (30 %). (Personal communication, April 26, 2012, Abdulbari Bener, former research professor of public health, Weill Cornell Medical College in Qatar).

Lacking information on the preferred recruitment strategies of persons speaking our target languages for psychometric testing of the MAI, the research team documented patient responses by: target language group; invitations to participate; preferences regarding follow-up in person or by post; preferred mode of follow-up reminders; and consent procedure.

The purpose of this paper is to share the research team’s strategies used to recruit and retain appropriate candidates for a study in four languages involving repeated survey administration among a multicultural, mosaic population in the Middle East, and consider implications of the outcomes of the used procedures so as to inform future research.

## Methods

### Design

This mixed methods case study conducted during psychometric testing of the MAI instrument, describes and evaluates the recruitment and enrollment strategies used with Arabic, English, Hindi, and Urdu speakers who participated. Data collection for psychometric testing involved two components. First, for concurrent validity testing, research assistants (RAs) distributed the MAI and a comparison instrument, the Vancouver Index of Acculturation (VIA) [40], as well as a demographics instrument. Second, 2–3 weeks later, the MAI was given a second time for reliability testing. The research team designed the MAI, a 25-item instrument, to measure individuals’ acculturation, and used for concurrent validity testing the VIA, a 20-item instrument [36]. The results demonstrating the validity and reliability are to be published elsewhere. Each instrument required 5–10 min to complete. The demographic instrument required 3–5 min. This project was approved by the human subjects review committees of Weill Cornell Medical College in Qatar, Hamad Medical Corporation (HMC), and the University of Michigan.

### Setting

The study was conducted in January through June 2012 in two types of ambulatory care settings in Doha, Qatar: a) the HMC Internal Medicine outpatient clinics which are located at the main hospital, and b) the Primary Healthcare Centers (PHCs) that are open most of the day and have appointment and walk-in based clinics. Six of 13 PHCs located in Doha [41] were assigned to the research team as recruitment sites by the Primary Health Care Corporation (PHCC), as these sites had large, diverse patient populations [19]. These centers were Al Rayyan, Madinat Khalifa, West Bay, Omar Ibn Al Khattab, Abu Baker Saddiq, and Al Gharrafa.

### Participants

Adult patients and their family members or friends who were visiting a research site during the recruitment process were invited to participate if they spoke Arabic, English, Hindi, or Urdu as a primary language.

### Study procedures

#### Recruitment and consent procedures

##### Recruitment for the baseline survey conducted for concurrent validity testing

For the initial recruitment, the trained RAs, who were fluent in English plus at least one other target language, approached potential participants in the clinic waiting areas. During preparation for data collection the research team developed a “cultural clues model of recruitment” that involves the RA using cultural clues to help optimize and standardize the strategies for successfully identifying and recruiting eligible participants for the four target populations. The RAs approached and recruited face-to-face participants who appeared to be from their own culture and one of the target language groups on the basis of cultural clues, like the patient’s style of dress, scent when present, or other intuitive identifying patterns or trends. The RAs explained in the target language the purpose of the study and assessed each person’s interest in participating. The RAs then assessed the person’s eligibility according to the inclusion and exclusion criteria listed in Table 1 (below). They read aloud or had the study enrollee read to her or him a copy of the waiver of signed consent in the participant’s preferred language, and a copy of that document was given to the patient [4, 22, 23, 25, 27]. No compensation was offered to participants for taking the baseline survey for concurrent validity testing.

**Table 1:** Inclusion and Exclusion criteria

Inclusion criteria	Exclusion criteria
•Speaks (and reads, if preferring to self-administer the baseline survey) the target language as the first or a primary language^a^ •Verbally consents to participate •Aged ≥18 years	•Not interested/declines participation •Has a severe, debilitating illness that precludes meaningful participation •Does not speak the study language as a primary language •Has low literacy in the study language (for postal follow-up only)

^a^ Primary language was defined as a language the person grew up speaking or reading from childhood or as determined by sociocultural norms, such as the work environment of one’s home country

##### Recruitment for the follow-up survey needed for reliability testing

Targeted recruitment was used to fulfill a goal of ten completed in-person follow-up interviews per language, with the interviewees in each language group divided about equally by gender and literacy status. Compensation for participation in the form of a prepaid phone card (Hala Card of 100 Qatari Riyals, about $30 USD) [2, 4, 34], was offered to persons invited to participate in face-to-face interviews to compensate for the time and cost of coming to the hospital to complete the interview. The literate participants at baseline who were not assigned to the in-person follow-up were offered participation by post. Illiterate participants were not offered participation in reliability testing as the team was not sure who would fill out mailed forms. We did not compensate post-participants because: they received a self-addressed, stamped envelope; there was no cost of travel to the clinic as incurred by face-to-face participants; and foremostly, budgetary constraints were prohibitive. To reflect the cultural diversity of primary English speakers, the research team recruited participants from Europe, the Philippines, Indian subcontinent, and Arab countries. For each language group, recruitment continued until the minimum *n* = 100 quota for all languages was reached. Each participant was assigned a unique numerical identifier matching labels on the participant’s survey materials. RAs were trained to collect qualitative field notes that included the ease or difficulty of recruiting a specific language group or literacy level and any voluntarily shared information on reasons for declining participation. The RAs also recorded each participant’s self-reported cultural background and their preferred mode of contact for follow-up reminders. All approached individuals who declined participation were thanked and no further information was asked of them.

#### Follow-up procedures

##### Follow-up in person

If the participant agreed to an RA’s invitation to follow up in person, the RA scheduled a meeting date and time about 2–3 weeks in the future and reminded the participant of the appointment using the participant’s preferred means of communication (phone, text message, or e-mail). Participants were contacted 1–2 days before the appointment, with a maximum of three reminders given to participants who could not be reached.

##### Follow-up by post

If the participant agreed to follow up by post, s/he was asked to write her/his postal address on an envelope, that was sent to her/him 10 days later with the second MAI survey (for reliability testing) and a stamped (prepaid) envelope addressed to the research office; surveys and return envelopes were coded for matching purposes.

As a control to determine the duration a study packet would spend in the postal system, the research team mailed two participant packets to RAs using different addresses. In the pilot data, the time spent en route was 5 days in 1 instance and 14 days in the other (mean, 8.5 days). On the basis of this pilot data, the research team issued a first reminder about mailing back the completed follow-up survey on the fifth day after packages were mailed out. Contact was made using the communication mode (phone, text, e-mail) chosen by participants at baseline; a maximum of three reminders were given to participants who could not be reached [12]. The research team recorded details of all aspects of data collection.

### Statistical analyses

The status of recruitment was summarized and descriptive statistics were used. The status of survey completion, consent forms, participants’ preferred mode of reminders, and the participants’ addresses were collated by language group. Chi-squared tests (or Fisher exact tests, if the sample sizes in the categories were too small) were used to test for significance. If a significant difference was observed, a multiple chi-squared (or Fisher exact) test was conducted, comparing two languages at a time. If the sample size in the categories was too small, the multiple comparison tests were not conducted and differences by language group were assessed according to percentages only.

Sample characteristics are summarized by type of follow-up (in person or by post). Chi-square test or Fisher exact test as appropriate was used for: (a) participants who agreed to in-person follow-up, to test for any significant difference between those who did and those who did not follow up; and (b) participants who agreed to follow up by mail, to test for any significant differences among the four groups: persons whose address was unusable, those who provided no address, persons whose survey was mailed out and delivered but never mailed back, and those with a completed, mailed-back survey. If a significant difference was observed, the same method of multiple comparison testing described above was used. A *p*-value of less than 0.05 was considered significant. All analyses were done by using Stata/SE version 12.1 [42].

## Results

### Illustration of recruitment, enrollment and participation for psychometric testing of the MAI

**Fig. 1:**
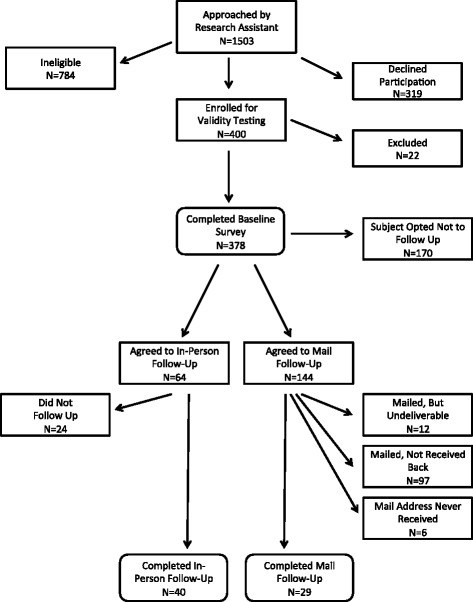
Recruitment scheme of participants

As illustrated in the Fig. 1, for the initial concurrent validity testing, RAs approached 1503 people who appeared to be from their own culture using the cultural clues model. Participant eligibility was then assessed according to the inclusion and exclusion criteria (see Table 1). Interested and eligible participants (*N* = 400) were invited to complete the baseline survey (the MAI, the VIA, and the demographic instrument) and to participate in a follow-up survey for reliability testing (MAI only) 2–3 weeks later. Among the 378 people that completed the baseline concurrent validity testing, 170 opted out of the follow-up survey for reliability testing. The follow-up involved a second administration of the MAI in person or by mail. Among the 64 that agreed to an in-person follow-up, 40 (63 %) participants actually completed the in-person interview. Among the 144 persons who agreed to a mail follow-up, 126 were successfully mailed to deliverable addresses (i.e., 18 were returned) and only 29 (20 %) participants completed and returned the follow-up survey.

### Response to recruitment by language group for the initial survey for concurrent validity testing

Among the 1503 potential participants approached by RAs, 596 (40 %) persons were excluded because the target language was not their primary language (Table 2). The proportions of participants misidentified for recruitment using the cultural clues model for the target Arabic, English, Hindi, and Urdu groups were 9, 41, 66, and 23 %, respectively. Of 719 persons meeting all inclusion criteria, 319 (44 %) declined an invitation to participate; the most common reasons offered by those declining were lack of interest, as cited by 125 persons (39 %), and limited time, as mentioned by 114 persons (36 %). Once a language group’s quota was met, persons approached about participation in that group were deemed ineligible to enroll; this exclusionary criterion applied to 152 participants. Of the 400 total participants, 22 persons were later excluded (one was younger than 18 years of age, and the others had incomplete recruitment data or surveys).

**Table 2:** Recruitment outcomes among potential participants approached for a baseline survey for concurrent validity testing according to target language group

Enrollment Status		Participants according to language group			
	Total of all languages	Arabic speakers	English speakers	Hindi speakers	Urdu speakers
	*N* = 1503	*N* = 309	*N* = 302	*N* = 563	*N* = 329
	n (%)	n (%)	n (%)	n (%)	n (%)
Ineligible	784 (52)	96 (31)	154 (51)	393 (70)	141 (43)
Not patient’s primary language	596	27	123	370	76
Quota full	152	66	24	22	40
Below age cut-off	15	2	3	0	10
Already took the survey	6	1	3	1	1
Reason not recorded	15	0	1	0	14
Declined	319 (21)	113 (37)	48 (16)	70 (12)	88 (27)
Not interested	125	72	14	14	25
No time	114	24	23	30	37
Sick/sick child	28	11	4	6	7
Family issue	11	0	2	4	5
Other	15	6	1	1	7
Unknown	26	0	4	15	7
Enrolled	400 (17)	100 (32)	100 (33)	100 (18)	100 (30)
Completed baseline survey	378	92	95	92	99
Excluded	22	8	5	8	1
Incomplete baseline survey	21	7	5 ^a^	8 ^a^	1
Below age cut-off	1	1	0	0	0

^a^ A Hindi-speaking participant had uncompleted Hindi and English surveys. He started the Hindi version but found it difficult, so switched to the English. Next, he had to see the doctor, and he never returned to finish

### Research assistant field observations about people opting out of the study

As noted, 319 participants of all approached declined the RAs’ invitations to participate in the study. The RAs’ field notes provide contextual information about reluctance to participate. For example, the RAs had difficulty successfully recruiting participants with low literacy skills in general; most people with low literacy, regardless of the target language group, typically did not want to participate. This finding was especially the case for Arabic-speaking men. Such was also the case with Hindi-speaking women with low literacy; some of them were housemaids who feared offending their sponsors, who usually accompanied them [20]. Most of the encountered female Arabic speakers who had low literacy were elderly and Qatari. Some declined to participate when they realized the survey content was related to culture. Of interest, some low-literacy Urdu-speaking participants were Pathan or Baloch and had been living in Qatar for a long time, possibly since birth. Next, a few of the participants with low literacy recommended that the RAs recruit an educated person instead, saying for example, “Go to someone who is educated,” or “Search [for] another one, who can read and write, because I cannot….” RAs observed that some participants marked a higher education level than what was apparent to the RAs. Other participants did not want to participate because they feared they might not understand the survey questions. Finally, some Arabic speakers seemed uncomfortable with the idea of filling out what they perceived as an “official document.”

### Participant reactions to providing informed consent

Participants’ preferences for administration of the informed consent and the baseline survey are shown in Table 3. The Arabic and English groups differed in their preference for the informed consent procedure; 68 % of the Arabic speakers chose to skip reading the consent form, whereas 96 % of the English speakers chose to read it by themselves (*p* < 0.001). In the Hindi and Urdu groups, similar proportions chose self-administered consent (62 and 63 %, respectively) versus RA-administered consent (35 and 38 %, respectively). In the Arabic, Hindi, and Urdu groups, most participants who opted to have the consent form read aloud to them (91–100 %) had a low literacy level, whereas none in the English group chose that option (*p* = 0.004). All participants in the English group chose to self-administer the baseline survey, whereas 23–40 % in each of the other groups requested RA assistance (*p* < 0.001).

**Table 3:** Participants’ chosen modes for consent procedure and baseline survey for concurrent validity testing by literacy status and language groups

Participants’ preferences (and literacy status)	All baseline respondents	Participants by language group				P value
	*N* = 378	Arabic	English	Hindi	Urdu	
	n (%)	*N* = 92	*N* = 95	*N* = 92	*N* = 99	
		n (%)	n (%)	n (%)	n (%)	
Chosen mode for informed consent						<0.001
Read consent form alone	228 (60)	18 (20)	91 (96)	57 (62)	62 (63)	
RA reads aloud	80 (21)	11 (12)	1 (1)	32 (35)	36 (36)	0.004
Low literacy	74	11	0	29	34	
Literate	6	0	1	3	2	
Skips reading	70 (19)	63 (68)	3 (3)	3 (3)	1 (1)	0.87
Low literacy	24	23	0	1	0	
Literate	46	40	3	2	1	
Chosen mode for baseline survey						<0.001
Self-administers	287 (76)	62 (67)	95 (100)	71 (77)	59 (60)	
RA reads aloud, fills in responses	91 (24)	30 (33)	0 (0)	21 (23)	40 (40)	

### Preferences for mode of follow-up reminders

As illustrated in Table 4, the most commonly preferred communication option for follow-up reminders, chosen by 144 of 208 participants (69 %), and notably, 61 of 63 Urdu speakers (97 %; *p* < 0.001 for comparison with the other language groups), was telephone call only. The next most popular option was e-mail only, chosen by 40 participants (19 %), half of them from the English group. Text messaging as the only mode of contact for reminders was favored by four participants (2 %), all of them Arabic speakers.

**Table 4:** Preferred mode for receiving reminders and preference for sharing address with the research team among individuals who agreed to participate in a follow-up survey for reliability testing ^a^

Participants’ preferred option	All participants agreeing to follow-up n (%)	Participants, according to language group, n (%)				P value
		Arabic speakers	English speakers	Hindi speakers	Urdu speakers	
		n (%)	n (%)	n (%)	n (%)	
Contact mode for follow-up reminders (N)	208	40	65	40	63	<0.001
Phone call	144 (69)	24 (60)	34 (52)	25 (63)	61 (97)	
E-mail	40 (19)	9 (23)	20 (31)	11 (28)	0	
Phone call & e-mail	12 (6)	2 (5)	8 (12)	1 (3)	1 (2)	
SMS text	4 (2)	4 (10)	0	0	0	
Phone call & SMS text	3 (1)	1 (3)	2 (3)	0	0	
None	5 (2)	0	1 (2)	3 (8) ^b^	1 (2) ^c^	
Provision of mailing address^f^ (N)	144	26	52	19	47	0.011
Given at baseline	126 (88)	24 (92)	49 (94)	18 (95)	35 (74)	
Asked RA to call later	17 ^d^ (12)	2 (8)	2 (4)	1 (5)	12 (26)	
Agreed to send later via e-mail	1^e^ (1)	0	1 (2)	0	0	

^a^ Due to rounding, percentages may not add up to 100 %. *SMS* short message service

^b^ Two participants wanted no reminders. A third participant provided no phone number or e-mail address; although she promised to call before her visit, she did not

^c^ One participant did not indicate a preference for receiving reminders or providing a postal address. When called for an address, she did not provide one; hence, she was classified with “none.”

^d^ Nine participants provided their postal addresses, 5 did not, and 3 provided wrong addresses

^e^ One participant promised to provide her postal address by e-mail. However, after being contacted twice by e-mail, she said that she could not provide it and was out of town

^f^ Pertains to participants who agreed to follow-up by mail

### Compliance with preference for in-person mode to take the follow-up survey for reliability testing

For the follow-up survey conducted for reliability testing the response rate was 63 % among participants who agreed to follow-up in person (40 of 64 participants), compared with 23 % among those agreeing to do so by post (29 of 126 participants) (Table 5). Among participants who accepted an invitation to follow up in person, nonresponse was highest in the Hindi-language group; RAs had to enroll 21 Hindi speakers who agreed to in-person follow-up to obtain 10 completed follow-up surveys. At baseline, majorities in the Hindi and Arabic groups chose not to follow up, whereas a plurality and majority of Urdu and English speakers agreed to follow-up by post (56–57 % vs. 47–55 %, respectively; *p* < 0.001).

**Table 5:** Follow-up status for reliability testing by language group among participants completing the baseline survey and according to the follow-up mode chosen at the time of baseline testing^a^

Decision at baseline for follow-up and actual follow-up outcome	All baseline respondents	Participants according to language group				
	*N* = 378	Arabic	English	Hindi	Urdu	P value
	n (%)	*N* = 92	*N* = 95	*N* = 92	*N* = 99	
		n (%)	n (%)	n (%)	n (%)	
Chose no follow-up	170 (45)	52 (57)	30 (32)	52 (57)	36 (36)	
Agreed to in-person follow-up	64 (17)	14 (15)	13 (14)	21 (23)	16 (16)	0.31
Followed up	40	10	10	10	10	
Did not follow up	24	4	3	11	6	
Agreed to follow-up survey by post	144 (38)	26 (28)	52 (55)	19 (21)	47 (47)	0.31
Not mailed out^b^	6	0	2^c^	0	4 ^d^	
Mailed out	138	26	50	19	43	0.89
Undeliverable	12	4	4	1	3	
Completed, mailed back	29	4	11	4	10	
Not received back	97	18	35	14	30	

^a^ Due to rounding, percentages may not add up to 100 %

^b^ No mailing address provided

^c^ One participant asked the research assistant to call later for the address, but she never provided it; she later asked to receive the survey by e-mail. Another participant promised to e-mail her address; however, after 2 e-mail reminders, she replied that she could not provide her address and was out of town

^d^ Two participants did not provide their address upon reminder. A third participant did not provide her street address and wanted to receive the survey by e-mail. The father of a fourth participant would not allow their address to be disclosed

### Compliance with preference for post to take the follow-up survey for reliability testing

Among 144 participants who agreed to follow up by post, 126 (88 %) provided their mailing address. Of the remaining 18 participants, one person promised to e-mail her address later to the RA, but subsequently she said she could not do so; the rest (17 participants, 12 (67 %) of them Urdu speakers) asked the RA to call them for it later by phone. When reached by telephone later as requested, nine participants (53 %) provided an apparently valid address, five participants (29 %) provided no address, and 3 participants (18 %) gave an incorrect address. Ultimately, among the 138 surveys mailed out by the research team, 12 were undeliverable; 29 follow-up surveys were completed and mailed back (23 %). Among persons whose surveys were mailed out to a deliverable address, the nonresponse rate did not differ significantly by language group: 18 of 22 Arabic speakers (82 %), 14 of 18 Hindi (78 %), 35 of 46 English (76 %), and 30 of 40 Urdu (75 %) (Table 5). Among persons agreeing to follow up by post, the proportions choosing to provide their mailing address at baseline rather than asking to do so later by telephone differed between the Urdu speakers and the other groups (74 % of Urdu speakers vs. 92–95 % of others provided at baseline and 26 % vs. 4–8 %, respectively, asked to give later by phone; *p* =0.011) (Table 5).

### Comparison of the demographics of all people who agreed to participate in the follow-up survey for reliability testing

The majority of participants were relatively young; 40 % were 25–34 years of age and 26 % were 35–44 years. A plurality of the literate participants, 41 %, were college graduates. Half of all the participants had been living in Qatar for no more than 5 years. Islam was the most commonly reported religion among participants (66 %), followed by Christianity (20 %). Among the participants agreeing to postal follow-up, a borderline significant difference was found between genders; more women than men did not disclose their postal address (83 % vs. 17 %) and more men than women (76 % vs. 24 %) responded at follow-up (*p* = 0.045) (Table 6). A significant association was also found by area of ancestry; the proportion of participants not mailing back a completed follow-up survey or providing an undeliverable address was higher in participants of African ancestry than in persons of European ancestry. Otherwise, no significant differences were found related to age, level of education, years lived in Qatar, or religion.

**Table 6:** Demographic characteristics of participants who did and did not complete follow-up surveys, whether by post or in person

Variable	Agreed to in-person Follow-up, *n (%)* ^a^		*P* value	Agreed to postal follow-up, *n (%) a*				*P* value
	Followed up (*n* = 40)	Did not follow up (*n* = 24)		Survey completed, mailed back (*n* = 29)	Survey not mailed back (*n* = 97)	Undeliverable (*n* = 12)	Address not available (*n* = 6)	
Gender			0.40					0.045
Male	21 (53)	10 (42)		22 (76)	56 (58)	7 (58)	1 (17)	
Female	19 (48)	14 (58)		7 (24)	41 (42)	5 (42)	5 (83)	
Age			0.92					0.099
18–24 years	4 (10)	3 (13)		0	13 (13)	1 (8)	1 (17)	
25–34 years	14 (35)	10 (42)		10 (34)	38 (39)	8 (67)	4 (67)	
35–44 years	12 (30)	7 (29)		8 (28)	24 (25)	3 (25)	0	
45–54 years	4 (10)	3 (13)		4 (14)	16 (17)	0	1 (17)	
55–64 years	4 (10)	1 (4)		6 (21)	5 (5)	0	0	
65–74 years	2 (5)	0		1 (3)	1 (1)	0	0	
Education			0.93					0.23
No formal education	3 (8)	1 (4)		0	0	0	0	
Primary school ^b^	4 (10)	3 (13)		0	1 (1)	0	0	
Middle school^b^	4 (10)	1 (4)		1 (4)	3 (3)	1 (10)	0	
High school^b^	10 (25)	8 (33)		2 (7)	24 (25)	2 (20)	1 (25)	
Some college	15 (38)	8 (33)		11 (38)	45 (46)	5 (50)	1 (25)	
College graduate	4 (10)	3 (13)		15 (52)	24 (25)	2 (20)	2 (50)	
Ancestral region			0.64					0.012
Southern Asia	21 (49)	17 (71)		15 (50)	58 (59) ^c^	4 (33)	4 (57)	
Southeastern, East Asia^d^	4 (9) ^c^	2 (8)		0	5 (5)	2 (17)	1 (14)	
Western Asia	4 (9)	1 (4)		4 (13)	12 (12) ^c^	2 (17)	0	
Europe	6 (14) ^e^	1 (4)		10 (33) ^f^	9 (9) ^f^	0	1 (14) ^c^	
Africa	7 (16)	3 (13)		1 (3)	11 (11)	3 (25)	0	
The Americas	1 (2) c	0		0	4 (4) ^g^	1 (8)	1 (14) ^c^	
Years lived in Qatar			0.73					0.44
0–5	20 (50)	14 (58)		15 (52)	47 (48)	6 (50)	3 (50)	
6–10	4 (10)	1 (4)		3 (10)	10 (10)	3 (25)	2 (33)	
≥11	16 (40)	9 (38)		11 (38)	40 (41)	3 (25)	1 (17)	
Religion			0.50					0.21
Muslim	27 (68)	19 (79)		19 (66)	61 (63)	8 (67)	4 (67)	
Hindu	1 (3)	2 (8)		1 (3)	9 (9) ^f^	0	0	
Christian	8 (20)	3 (13)		4 (14)	23 (24)	3 (25)	1 (17)	
Other	2 (5)	0		4 (14)	4 (4) ^g^	1 (8)	0	
Preferred not to share	2 (5)	0		1 (3)	0	0	1 (17)	

^a^ Due to rounding, percentages may not add up to 100 %

^b^ Had some schooling at or completed that level of education or the equivalent

^c^ Participant had additional background

^d^ One participant was from Australia and New Zealand

^e^ Three participants had additional background

^f^ Two participants had additional background

^g^ One participant was from Polynesia

## Discussion

There are significant challenges to successfully recruiting appropriate research candidates in extremely high-density, multicultural populations. When initiating this study, there was no identifiable literature informing cultural and social norms relative to recruitment in highly diverse populations. Based on this research, substantively more information is available to guide investigators engaged in survey research.

### Cultural clues model

For the initial recruitment for concurrent validity testing, the research team developed and utilized a “cultural clues model of recruitment” that leveraged the RAs’ affiliations with the target communities. An innovative approach, the trained bilingual RAs, using a cultural clues model developed by the researchers, accurately identified 60 % of the persons approached—usually persons from their own culture—about recruitment to one of the study language groups (Arabic, English, Hindi, Urdu). In extremely diverse multicultural settings with so many languages, researchers need some mechanism of recruiting subjects, and while not perfectly, this approach worked satisfactorily. The research team found the model was helpful in standardizing the recruitment approach with particular target groups and in navigating uncertain social rules of engagement by gender and age group. The heterogeneity among the target language populations posed a particular challenge [1]. For example, the RA might have correctly identified the cultural background of the person as Indian, but given India’s diversity of spoken languages, the person did not speak Hindi. Moreover, while spoken Hindi can be learned conversationally, learning written Hindi typically requires formal study; thus, some participants preferred to take the written survey in English, despite their oral fluency in Hindi. With English as the lingua franca in Qatar, the English group particularly comprised participants of various cultural backgrounds. Traditional Islamic clothing, like the neghab (face veil, also known as niqab), can make it difficult to pinpoint a person’s culture or language. Nonetheless, the RAs were able to successfully identify accurately just under half of the total approached as they were affiliated with the target language and culture. The actual percentage likely is higher because the research team knew the RAs correctly identified the culture of some participants, e.g., Indian, but the subject spoke a different language than Hindi, e.g., Malayalam.

On the flip side, 40 % of approached individuals could not be enrolled because they didn't speak one of the four study languages. As the RAs were trained to approach any individuals who they thought might be eligible to participate in order to meet recruitment goals as quickly as possible, the research team still believes the model worked remarkably well. The RAs were best at identifying Arabic and Urdu speakers. Hindi language participants were the most challenging group for the RAs to recruit, though this was attributable in part to the relatively large number of people who are culturally Indian but preferred English to Hindi. English speaker recruitment was also challenging, though this is not so surprising given the highly heterogeneous nature and diversity of individuals in Qatar who use English as their primary mode of communication.

Over 44 % of fully eligible persons declined participation, and RAs heard from persons the most common reasons to be lack of interest or time. While these may have been the stated reasons, there may have been other contributing factors as well. Based on previous research, [26] challenges faced in recruitment could be attributed to fear or stigma. These concerns would be compounded by a lack of awareness of the community about research in general.

Among those choosing to participate, there were interesting variations in preferences relative to disclosing contact information. Some participants were hesitant to disclose their mailing address, while others requested to be contacted by telephone for that information (as home addresses are infrequently used, this was an understandable response) [16, 26]. Unknown cultural reasons may explain some participants’ nondisclosure; for example, one participant mentioned that her father would not allow her to reveal their postal address [31]. Moreover, people are strongly disinclined to refuse others’ requests; thus, sometimes a participant’s asking the RA to call later for the address may have been an indication that she/he was not interested in participating [25]. In some cases, the address may correspond to a property owned by a participant’s spouse, parent, or even a family member’s employer, thus necessitating a conversation first (in particular, wives with their husbands) to seek permission [20, 21, 26, 27].

These findings are consistent with Hall’s anthropological theory published in 1976 about high and low context societies [43]. Arab culture has been characterized as a high context with an emphasis on the collective over individual priorities, and as having a high sense of social stability, and slow pace of societal changes [44, 45]. Consequently, approaching people outside of accepted social ‘scripts’, asking questions with unknown social consequences, lack of trust and awareness about research—despite the RAs’ assurances—might have lead to reluctance to disclose information. The difficulty faced by the RAs is consistent with the premise that social interactions typically are scripted (particularly public ones) in high context Arab societies such as in Qatar, and can hold very different social implications than they do in low context, individualistic societies as in most Western societies.

### Variations in response to preference for providing informed consent

Reading a study-information sheet in lieu of providing signed, informed consent revealed interesting differences: two-thirds of primary Arabic speakers chose to skip reading the consent form, whereas virtually all participants in the English group preferred to read it themselves [22]. This finding from the Arabic speakers could be explained by a high level of trust, a lack of concern about dangers, unawareness of one’s rights concerning participation in research [30], a lack of interest, illiteracy, or it could be explained by other reasons. This would also be consistent with high context behavior once trust was established with the same culture Arabic RAs. An alternate interpretation of the high rates of the Arab subjects preferring the RA to read the consent could be a complete lack of trust in the process, with no intention of participation. The potential Arab participants at the same time might have felt ‘trapped’ by social norms that discourage causing others to lose face, by rejecting their advances in public. In this context, the relatively high levels of loss to follow up and ‘deception’ in providing addresses could be seen as culturally acceptable responses to an ambiguous social situation. The high level of interest in the consent form among the heterogeneous English group could indicate research acculturation—that is, recurrent experiences over time with research in various settings, a feeling of compulsion to read such documentation, or a higher level of education, among other possible reasons. These interpretations aside, nearly a quarter of the participants chose to have the RA read the baseline instruments to them aloud, demonstrating the feasibility of recruiting and enrolling research participants with low literacy in an extremely high-density, multicultural setting like Doha, Qatar, a setting with residents from all over the world.

### Responses to invitation for the follow-up survey for reliability testing

This is the first known research documenting actual survey response rates for follow-up in person or by post in Qatar and more broadly, the Middle East. More than half the Arabic- and Hindi-speaking groups declined follow up by either format. This could be due to a lack of time, no postal address, lack of availability, transportation issues, or possible feelings of discomfort [3, 6, 16, 23, 25–27, 29]. The response rate was higher for in-person follow-up than for follow-up by post [28]. Due to budgetary constraints, the research team could only offer compensation to participants who returned to the hospital for face-to-face interviews. It is tempting to attribute this difference to the compensation provided to participants who came back for completion in person. However, the amount provided, 100 Qatari Riyals amounted to be little more than the incurred transportation and time costs. While complicating interpretation of response rates compared with post participants, this choice was justified as the only cost to the postal group was the brief time to complete the instruments and to mail the pre-paid, stamped envelope. Although compensation for participation can serve to motivate participants [2, 4, 6, 27, 34], there is limited information on perceptions of compensation for research participation among extremely high-density multicultural populations [26, 27].

Cultural factors may have impacted the results as well. For example, asking for addresses or contact information should not be assumed to be culturally neutral. Asking questions that are neutral in one culture, such as a request for an address, might be fraught with difficulty in another, particularly among women, who may have different cultural expectations of probity. Moreover, the very act of approaching another in a public place and asking questions that have unknown social consequences may be far more significant than in Western countries, and may represent a barrier to participation in research in the Arabian Gulf Region. As noted in this study, among older Qatari men and women, rates of even completing the initial screen were very low. Arab culture is a very high context culture,- therefore approaching people outside of accepted social ‘scripts’ may well have resulted in a polite excuse to avoid what was perceived to be a possible contravention of social etiquette by the researchers.

The most popular mode of contact for follow-up reminders was a telephone call, followed by e-mail, although each group had its own preference [14, 15, 32]. Surprisingly, text messaging was typically not the reminder mode of choice, even though it is a widely used means of communication in Qatar and worldwide. Unlike other studies [8, 10, 14], we observed in all four language groups a poor response rate for follow-up by mail, a rate that is lower in fact than those reported by many other international studies [8, 12, 33, 34]. The poor follow-up rate could be due to the early infancy of research awareness in Qatar [30, 35], though this wouldn’t explain higher rates of in-person follow-up. An alternate explanation is the traditional lack of postal delivery to Qatari residents’ home addresses. Lack of compensation for postal follow-up could have been a factor. However, participants’ received a prepaid, stamped envelope in their follow-up packet, so financial burden was likely not a factor among most non-responders. Perceived burden of the act of mailing back the envelope could also have contributed. Compared with follow-up by post, in-person follow-up may be faster, and elsewhere has also found to be associated with greater adherence [8].

Over all, the majority of adults in our study were relatively young, well educated, and mainly Muslim or Christian; no statistically significant differences were found between the in-person and postal follow-up groups. However, among the postal follow-up participants, all of whom were literate, we found a borderline significant difference by gender. Specifically, the response rate for postal follow-up was higher among men than women, which could be explained by men’s easier access generally to postal services through their workplace. Some women were reluctant to share their postal addresses; in certain cases, the participant first needed to secure permission from her husband or parents [26].

The RAs had tremendous difficulty recruiting persons with low literacy [18], especially for the Hindi group. This can be explained at least partly by Qatar’s disproportionately high recruitment of well educated professionals to join its workforce; persons in Qatar with low literacy are mainly male workers who may be unable to sponsor their wives and families to travel to Qatar or to secure residency permits. In addition, we have observed in general a lack of Arabic-speaking workers with low literacy in Qatar; also, such persons seem to visit the health centers infrequently.

Some potential participants declined to enroll in the study when they realized that the survey topic was culture. This could be due to a belief among some that “culture” in Arabic is a subject for discussion only by cultivated, highly educated persons.

### Limitations

Generalizability is not a critical issue for interpretation of findings, as the primary purpose is to shed the light on challenges that might face researchers in studies conducted in a multicultural context in the Arabian Gulf region and expand understanding about poor response to mail follow up previously reported [21, 23, 27, 29]. Still, there are potential limitations to this study. Caution should be exercised in generalizing findings from our study, in part because the recruitment of participants for the target language groups involved the use of cultural clues that could be RA dependent to help identify participants meeting the criteria for study inclusion. As the ambulatory-care setting serves individuals of various social classes and backgrounds, participation rates could differ in more homogeneous settings; on the other hand, the inclusion of individuals from multiple backgrounds is a relative strength of this study and the cultural clues model enabled meeting recruitment goals. Unfortunately, primary alternatives to the cultural clues model would be approaching all subjects or using interval sampling. This would likely have been less efficient, more costly, and disruptive since Arabic, English, Hindi and Urdu would not be known by many potential participants, even though these are among the most common languages in Qatar. Also, persons with low literacy were excluded from postal follow-up for logistical reasons, as the team was unsure who would help such participants fill out their survey. The postal response rates might have been even lower if illiterate participants had been included. A larger study is needed with more participants for follow-up and inclusion of more persons of low literacy in face-to-face and postal follow-up to confirm whether the trends are the same.

## Conclusion

This study has implications for researchers interested in recruiting study participants in Qatar and the Arabian Gulf. The selection of RAs who are bilingual (speaking English and the target language), who understand the cultural backgrounds of the target populations, and who use cultural clues as an aid, seems to be the most effective recruitment strategy. There is variability among groups of different backgrounds in their preferred approach to the informed-consent procedure, and having RAs available to explain the study and read aloud any written study materials seems particularly important to the successful recruitment of participants with low literacy—a surprising number of participants preferred for the RA to read the document. Although in-person follow-up, including compensation for such participation, is a relatively expensive means of data gathering, it may improve the enrollment and response rates and help with gathering data from low-literacy populations, compared to follow-up surveys by mail. Use of the postal system in survey data gathering has genuine challenges that were not expected, e.g., addresses may be difficult to obtain, responses might be slow in coming, and respondents may comprise mainly men with a high level of literacy. Response rates by mail were notably low, and it is unknown if compensation, e.g., inclusion of a Hala card in the recruitment package, would have improved response rates. For follow-up reminders, telephone calls may be preferred over texting. The investigators hope these findings will assist researchers planning research in the Arabian Gulf region on key aspects such as budget preparation, timelines, training RAs, and allocating human- and other-resources.

## Abbreviations

MAI: Multicultural assessment instrument
VIA: Vancouver index of acculturation
RA: Research assistant
PO: Post office
PHCC: Primary Health Care Corporation
PHCs: Primary healthcare centers
